# Backwards and Bilateral: An Unexpected Variation of the Palmaris Longus

**DOI:** 10.7759/cureus.99053

**Published:** 2025-12-12

**Authors:** Soham Apte, Noah R Mitchell, Ian Kania, Uchenna Uduma, Abayomi G Afolabi

**Affiliations:** 1 Basic Sciences Department, University of Pikeville - Kentucky College of Osteopathic Medicine, Pikeville, USA

**Keywords:** bilateral palmaris longus, cadaver dissection, carpal tunnel syndome, forearm anatomical variation, median nerve compression, palmaris longus tendon, reversed palmaris longus

## Abstract

The palmaris longus (PL) is a slender forearm muscle that contributes to wrist flexion and tensioning of the palmar aponeurosis, though its presence and configuration can vary among individuals. This study describes a rare occurrence of bilateral reversed palmaris longus (RPL) identified during routine cadaveric dissection, in which the muscle belly is positioned distally, and the tendon extends proximally. Such a configuration has the potential to alter wrist biomechanics and may increase the risk of nerve compression syndromes, including carpal tunnel syndrome. The study highlights the clinical significance of RPL, particularly in surgical situations where tendon grafting or carpal tunnel release could be affected by the reversed anatomy, and emphasizes the importance of further investigation into PL width variations to better understand their relationship to forearm function and nerve compression risk.

## Introduction

The palmaris longus (PL) is a slender, superficial muscle found in the anterior compartment of the forearm. It originates from the medial epicondyle of the humerus and typically inserts into the palmar aponeurosis, contributing to wrist flexion and tensioning of the palmar aponeurosis [1,2]. However, despite its seemingly straightforward anatomy, the PL muscle has long been well-known for its remarkable variability [3-6]. This variability encompasses a spectrum of presentations, ranging from the complete absence of the muscle to variations in its form, including duplication, bifid tendons, and reversed configurations. While its absence in approximately 15% of the global population is well-documented, other variations in its morphology are less common and, consequently, less understood [7,8]. Among these rare variations, the bilateral reversed palmaris longus (RPL), where the muscle belly is distal to the tendon, represents a particularly interesting case, especially when presented bilaterally [6,9-11]. Estimates vary widely on the frequency of any occurrences of this, from as low as 0.5% to as high as 12% in some populations [6,12].

The PL muscle, despite its limited functional significance in human anatomy, has gained considerable importance in reconstructive surgery. This paradox is primarily attributed to two key anatomical characteristics: its superficial location within the forearm and its slender distal tendinous portion. These properties render the PL an ideal candidate for harvesting in various surgical procedures, particularly those requiring tendon grafts [13,14]. A point of clarification is warranted with respect to the term “inverted” when referring to this anatomical variation [15-17]. This term is less frequently applied when describing a distal muscle belly of the PL that is not directly connected to the palmar aponeurosis, with some authors suggesting reserving the term “reversed” for cases in which the muscle belly directly inserts into the palmar aponeurosis defined by Still and Kleinert [18]. For this study, we follow Still and Kleinert's definition [18].

While most individuals with PL variations remain asymptomatic, certain anatomical differences - particularly the reversed configuration - have been associated with clinical complications. These may include median or ulnar nerve compression, leading to symptoms such as pain, paresthesia, and muscle weakness [19]. The abnormal positioning of the tendon and muscle in RPL can also pose challenges during tendon grafting procedures, impacting surgical planning and technique [20]. Therefore, a comprehensive understanding of these variations is crucial for surgeons and clinicians, especially in preoperative planning, accurate diagnosis of forearm conditions, and effective management of potential complications [21].

The occurrence of bilateral RPL is an exceedingly rare anatomical finding, with only 55 cases documented in the literature from 1916 to 2018 [11]. This rarity underscores the importance of reporting and analyzing such cases to enhance understanding of PL muscle variations and their clinical implications [11]. This study presents a detailed anatomical description of a cadaver with bilateral RPL, identified during routine anatomical dissection. By contributing to the existing body of knowledge on PL variations, this study aims to emphasize the clinical significance of such findings, particularly in the context of nerve compression, surgical procedures, and potential complications.

## Materials and methods

This study was conducted in the anatomy laboratory at the University of Pikeville - Kentucky College of Osteopathic Medicine (UP-KYCOM) using 26 cadavers (52 upper limbs) available for educational dissection. A total of 28 cadavers were initially available; however, two cadavers were excluded because they lacked a PL muscle bilaterally, a well-documented anatomical variation, and therefore could not contribute measurement data. Demographic information (age, sex, ethnicity) was not provided to student dissectors per institutional policy and was therefore unavailable for this study. During routine dissection, the presence or absence of the PL was recorded in all cadavers. When present, the width of the PL muscle belly was measured bilaterally using a standardized protocol to establish baseline morphometric data for comparison. One male cadaver demonstrated a bilateral RPL, in which the muscle belly was located distally with a proximally oriented tendon. Measurements of this variant were compared against the compiled dataset of normal PL muscle belly widths.

A routine dissection following Grant’s standard approach to the anterior forearm was performed on the cadaver with the bilateral RPL [22]. The cadaver was procured for gross anatomy training from the Anatomical Gift Program (Dayton, OH) and had been embalmed within 24 hours of death in a formalin-based fixative solution [22]. The cadaver was positioned supine with both forearms in supination. An incision was made along the anterior aspect of the forearm, from the cubital fossa to the wrist [22]. The antebrachial fascia was carefully removed using both blunt and sharp dissection techniques to expose the underlying muscles [22]. The cephalic and basilic veins were preserved, while other superficial veins were removed for better visualization [22]. Measurements of the muscle belly width were taken using an electronic digital caliper (Mitutoyo Corporation, Kawasaki-shi, Kanagawa, Japan). The length of the muscle belly was measured from its most proximal to the most distal visible muscle fiber, and the width was measured at the midpoint of the muscle belly. Photographs were taken throughout the dissection process to document the findings visually.

## Results

**Figure 1:**
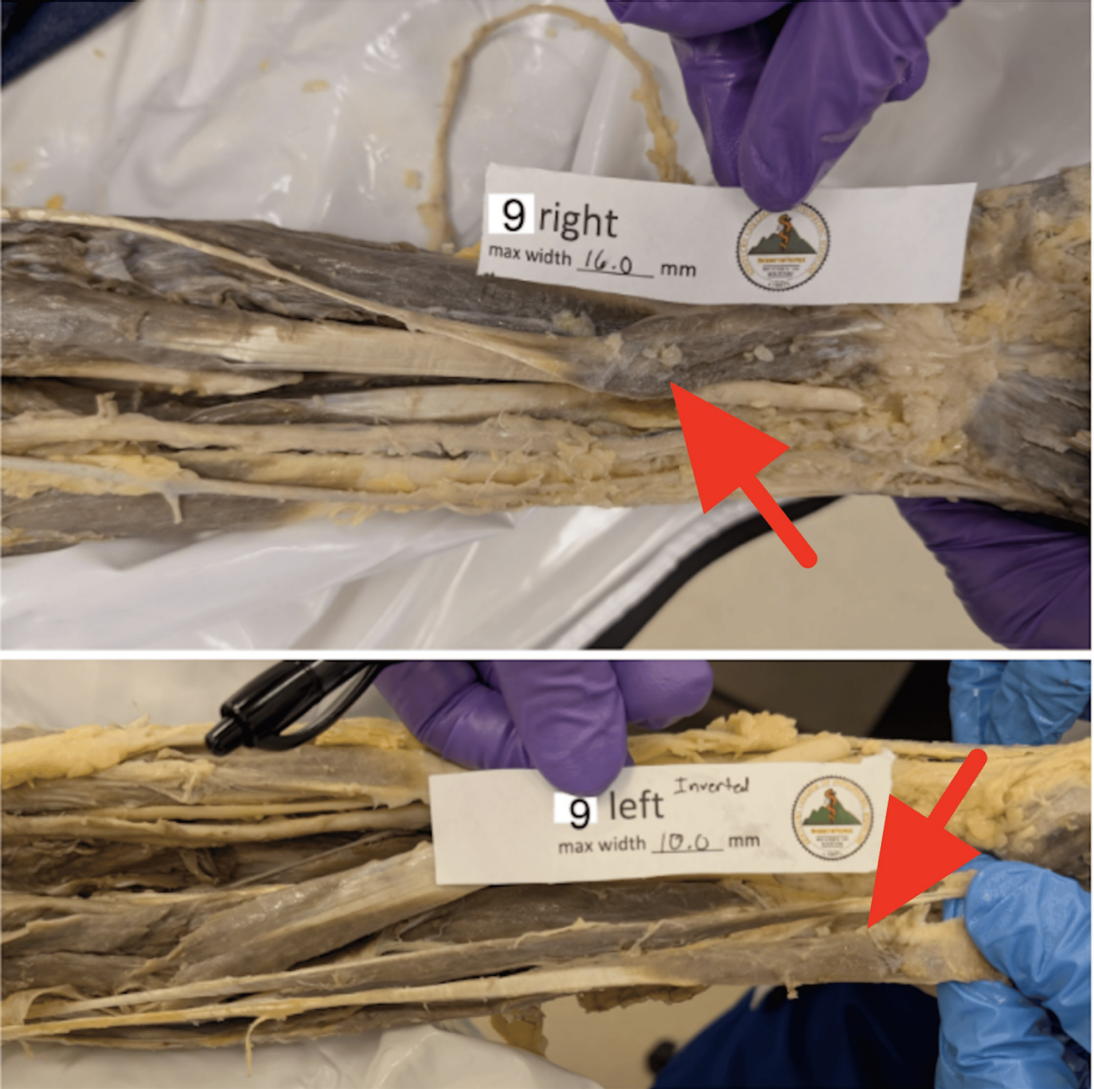
Identification of a bilateral RPL muscle with distal muscle belly (indicated by the red arrow) and proximally directed tendon during dissection of the superficial anterior porearm muscles RPL: reversed palmaris longus

Upon examination of the forearm muscles, the superficial layer of the muscles of the anterior compartment was exposed and identified, originating from the common flexor origin. The pronator teres, flexor carpi radialis, and ulnaris were easily identified. A tendon was also identified arising from the origin. Unlike the typical PL muscle, its belly was situated distally near the wrist, while its tendon ran proximally (Figure 1).

The long, thin tendon of this reversed muscle extended between the bellies of the flexor carpi radialis and ulnaris muscles. When traced distally, a muscle belly was located about 4 cm proximal to the distal attachment via a distal tendon attached to the anterior aspect of the flexor retinaculum palmar aponeurosis, confirming the presence of an RPL on both sides. The tendon and muscle belly were cleaned to facilitate clear visualization and documentation.

**Figure 2:**
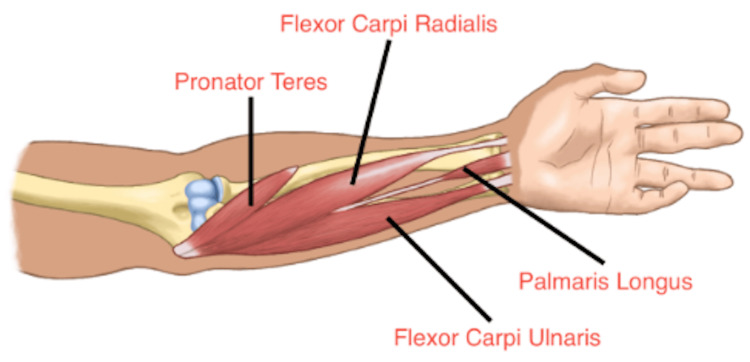
Bilateral presentation of an RPL muscle: an original illustration documenting the anatomical variation observed in both forearms RPL: reversed palmaris longus

The variation was observed on both forearms, and measurements were taken bilaterally. All findings were documented both in writing and through photographs. An original image was created to highlight the anatomical variation of an RPL muscle in this cadaver (Figure 2).

The normal PL widths from the 26 cadavers served as a reference baseline for identifying anatomical variation. In this sample, the right PL demonstrated an average width of 14.09 mm (±4.13 mm) and an interquartile range (IQR) of 6 mm, while the left PL averaged 13.53 mm (±3.87 mm) with an IQR of 7 mm. These values reflect the expected range of variation in muscle belly width among non-reversed PLs. In the cadaver with a reversed PL, the muscle belly measured 16 mm on the right and 10 mm on the left, resulting in a bilateral difference of 6 mm. Bilateral differences of this magnitude were also present among non-reversed PLs in the dataset, with some cadavers demonstrating differences of up to 7 mm. Although the individual widths of the reversed PL fall within or near the numerical ranges observed in the reference sample, they represent a distinct structural configuration compared with standard PL anatomy (Table 1).

**Table 1:** Comparison of normal PL muscle widths with bilateral measurements of an RPL, highlighting anatomical variation and discrepancy PL: palmaris longus; RPL: reversed palmaris longus

	Cadaver #	Right	Left	
2024	1	10.40 mm	10.50 mm	
	2	N/A	10.40 mm	
	3	4.00 mm	9.00 mm	
	4	7.00 mm	10.20 mm	
	5	10.00 mm	N/A	
	6	15.00 mm	16.00 mm	
	7	10.00 mm	8.00 mm	
	8	10.50 mm	10.00 mm	
	9	16.00 mm	10.00 mm	reversed
	10	10.20 mm	13.00 mm	
	11	18.00 mm	17.00 mm	
	12	22.00 mm	18.00 mm	
	13	15.00 mm	22.00 mm	
	14	9.00 mm	10.00 mm	
	15	N/A	22.00 mm	
	16	18.00 mm	11.00 mm	
	17	11.00 mm	10.00 mm	
	18	N/A	N/A	
	19	11.00 mm	11.00 mm	
	20	N/A	17.00 mm	
	21	N/A	10.00 mm	
	22	N/A	16.00 mm	
	23	16.00 mm	13.00 mm	
	24	14.00 mm	13.00 mm	
	25	N/A	11.00 mm	
	26	17.00 mm	18.00 mm	
	27	N/A	N/A	
	28	17.00 mm	18.00 mm	

## Discussion

The morphometric findings of this case provide important context for understanding the anatomical significance of a bilateral RPL. The 6 mm difference in muscle belly width between sides falls toward the upper range of asymmetry observed in the reference sample, in which some non-reversed PLs demonstrated bilateral differences of similar magnitude. Although this degree of asymmetry is not unique, it raises the question of whether width discrepancies play a consistent role in the morphology of reversed PL variants or simply reflect individual variation. Establishing standardized width measurements in future anatomical studies will be essential to determine whether patterns of asymmetry are characteristic of RPL or occur independently of the reversal. Clarifying these relationships may help explain how differences in muscle belly size or distribution could influence the functional behavior and clinical presentation of the RPL.

Because anatomical differences of this type may translate to biomechanical consequences, the presence of a bilateral RPL may have notable clinical implications, primarily due to its potential impact on wrist biomechanics and nerve compression risk [23]. While the PL typically contributes minimally to wrist flexion, the altered positioning in RPL, where the muscle belly is distally located, could influence the tension applied to the palmar aponeurosis [23]. This altered biomechanics may affect wrist stability and grip strength, particularly in repetitive wrist and hand movements [21]. Cases of RPL have been documented in individuals with occupations requiring intense manual labor, where repetitive forearm flexion and extension could exacerbate any underlying biomechanical changes and lead to functional limitations or discomfort [11].

RPL is also associated with an increased risk of nerve compression syndromes, including carpal tunnel syndrome and Guyon’s canal syndrome [24]. The presence of an enlarged or hypertrophied distal muscle belly can add pressure within the forearm’s flexor compartment, compressing nearby neurovascular structures, such as the median or ulnar nerve [25]. This compression can result in symptoms such as forearm pain, numbness, or tingling in the fingers and palm, which are common presentations of median or ulnar nerve involvement [25]. RPL-induced nerve compression has been reported in athletes and manual laborers, where the repetitive stresses placed on the flexor compartment may exacerbate these symptoms, potentially leading to exertional compartment syndrome in severe cases [26].

In surgical contexts, awareness of RPL is crucial, particularly in procedures involving tendon grafts or carpal tunnel release [20]. The PL tendon is frequently harvested for reconstructive surgeries; however, the reversed orientation of the RPL may render it less suitable due to its shorter proximal tendon [20]. In carpal tunnel release or other forearm surgeries, an undiagnosed RPL could complicate the procedure, increasing the risk of iatrogenic injury to adjacent structures like the median nerve [11]. Surgeons are advised to use preoperative imaging, such as ultrasound, to assess the orientation and suitability of the PL tendon for grafting in patients suspected of having an RPL [21].

In the literature, there is a notable lack of reports that document the width of the PL, especially in cases of RPL [11]. This omission is significant because measuring muscle width could provide insights into hypertrophy or atrophy, which may influence both the function and clinical presentation of the PL [27]. In cases where the RPL is hypertrophied, the increased muscle mass could contribute to a higher risk of nerve compression within the flexor compartment, leading to symptoms such as pain, tingling, or weakness [21]. Without standardized width measurements, it is difficult to assess how often hypertrophy occurs in RPL or its true impact on nearby structures like the median and ulnar nerves. Further studies measuring width could help clarify whether hypertrophy is a common adaptation in RPL and how it may relate to symptomatic presentations, especially in individuals with occupations requiring repetitive wrist flexion or gripping. Standardized width measurements could also aid in preoperative planning by providing benchmarks that help clinicians predict the likelihood of compression syndromes, improving diagnostic and treatment outcomes.

This study has several limitations that should be considered when interpreting the findings. The sample size was restricted to the 26 cadavers that met the inclusion criteria, as two specimens lacked a PL and could not contribute measurements. This limited number reduces the generalizability of the morphometric trends observed. Additionally, demographic information such as age, sex, and ethnicity was not available due to institutional policy, preventing any population-level or sex-based comparison that might influence PL morphology. The absence of clinical histories also limits the ability to correlate anatomical findings with functional symptoms, occupational patterns, or nerve compression risk. Finally, because all measurements were taken from embalmed cadavers, tissue distortion inherent to fixation may slightly alter muscle dimensions compared to in vivo conditions. Despite these constraints, the documentation of a bilateral reversed PL and associated width measurements provides meaningful anatomical insight and establishes a foundation for future research with larger and more diverse samples.

## Conclusions

This study highlights a rare occurrence of bilateral RPL, an anatomical variation with meaningful implications for clinical practice and surgical management. The unique configuration of the RPL, featuring a distally located muscle belly and proximally oriented tendon, underscores the importance of recognizing such variants during diagnostic evaluation and operative procedures. Awareness of these anomalies is essential, as they may alter wrist biomechanics, contribute to nerve compression syndromes, or complicate tendon harvesting. Thorough preoperative imaging and consideration of anatomical variability are therefore critical to ensuring surgical safety and optimal patient outcomes. In addition, this study contributes to the limited body of literature documenting measurements of RPL muscle belly width. Standardizing such measurements could improve understanding of the prevalence of hypertrophy or atrophy in these variants and their potential effect on forearm function and nerve compression risk. By documenting this bilateral case and emphasizing the clinical value of muscle width assessment, this study supports the continued investigation of RPL morphology and its developmental, functional, and surgical relevance.

## References

[1] Humphry Humphry (1872). The disposition of muscles in vertebrate animals. J Anat Physiol.

[2] Dalley AF, Agur AMR (2025). Moore’s Clinically Oriented Anatomy, 9e. https://premiumbasicsciences.lwwhealthlibrary.com/book.aspx.

[3] Macalister A (1875). Additional observations on muscular anomalies in human anatomy (third series), with a catalogue of the principal muscular variations hitherto published. Trans R Irish Acad Sci.

[4] Schaeffer JP (1909). On the variations of the palmaris longus muscle. Anat Rec.

[5] Morrison JT (1916). A palmaris longus muscle with a reversed belly, forming an accessory flexor muscle of the little finger. J Anat Physiol.

[6] Reimann AF, Daseler EH, Anson BJ, Beaton LE (1944). The palmaris longus muscle and tendon. A study of 1600 extremities. Anat Rec.

[7] Fragiadakis EG, Papavassiliou N, Giannikas A (1978). Variations of palmaris longus. Handchirurgie.

[8] Thompson JW, McBatts J, Danforth CH (2005). Heredity and racial variation in the musculus palmaris longus. Am J Phys Anthropol.

[9] Giunta R, Brunner U, Wilhelm K (1993). Bilateral reversed palmaris longus muscle--a rare cause of peripheral median nerve compression syndrome. Case report (Article in German). Unfallchirurg.

[10] Salgado G, Cantín M, Inzunza O, Muñoz A, Saez J, Macuer M (2012). Bilateral reversed palmaris longus muscle: a rare anatomical variation. Folia Morphol (Warsz).

[11] Longhurst G, Stone D, Mahony N (2020). Bilateral reversed palmaris longus muscle: a case report and systematic literature review. Surg Radiol Anat.

[12] Natsis K, Levva S, Totlis T, Anastasopoulos N, Paraskevas G (2007). Three-headed reversed palmaris longus muscle and its clinical significance. Ann Anat.

[13] Georgiev GP, Iliev AA, Dimitrova IN, Kotov GN, Malinova LG, Landzhov BV (2017). Palmaris longus muscle variations: clinical significance and proposal of new classifications. Folia Med (Plovdiv).

[14] Cooper DW, Burns B (2025). Anatomy, Shoulder and Upper limb, Hand Palmaris Tendon. https://www.ncbi.nlm.nih.gov/books/NBK519516/.

[15] Murabit A, Gnarra M, Mohamed A (2013). Reversed palmaris longus muscle: Anatomical variant - case report and literature review. Can J Plast Surg.

[16] Ruiz Santiago F, Moyano Calvente S, Ruiz Sánchez F (2006). Keys for the diagnosis of inverted palmaris longus muscle by ultrasound (Article in Spanish). Radiologia.

[17] Granite G, Wind G, Leighton M (2021). Palmaris longus inversus muscle present bilaterally in an 82-year-old cadaver and unilaterally in a 68 year-old cadaver. FASEB J.

[18] Still J, Kleinert HE (1973). Reversed palmaris longus muscle. J Bone Joint Surg.

[19] Acikel C, Ulkur E, Karagoz H, Celikoz B (2007). Effort-related compression of median and ulnar nerves as a result of reversed three-headed and hypertrophied palmaris longus muscle with extension of Guyon's canal. Scand J Plast Reconstr Surg Hand Surg.

[20] Sunil V, Rajanna S, Gitanjali Gitanjali, Kadaba J (2015). Variation in the insertion of the palmaris longus tendon. Singapore Med J.

[21] Olewnik Ł, Wysiadecki G, Polguj M, Podgórski M, Jezierski H, Topol M (2017). Anatomical variations of the palmaris longus muscle including its relation to the median nerve - a proposal for a new classification. BMC Musculoskelet Disord.

[22] Detton AJ (2021). Grant’s Dissector, 17e. https://pa-core.lwwhealthlibrary.com/Book.Aspx?Bookid=2832&sectionid=0.

[23] Pai MM, Prabhu LV, Nayak SR, Madhyastha S, Vadgaonkar R, Krishnamurthy A, Kumar A (2008). The palmaris longus muscle: its anatomic variations and functional morphology. Rom J Morphol Embryol.

[24] Demir Cİ, Yaşar EK, Dursun B, Alagöz MŞ (2021). Reversed palmaris longus muscle with median nerve compression symptoms: a case report and review of the literature. Ann Plast Surg.

[25] Hashem M, Alatassi R, Narinder K, Emran F (2020). Hypertrophied reversed palmaris longus muscle (pseudotumor) of the forearm causing median nerve compression: a case report. J Med Case Rep.

[26] Martens MA, Moeyersoons JP (1990). Acute and recurrent effort-related compartment syndrome in sports. Sports Med.

[27] Twoon M, Jones CD, Foley J, Davidson D (2017). Reversed palmaris longus muscle: a report of two cases. Case Reports Plast Surg Hand Surg.

